# Low 25‐Hydroxyvitamin D in Primary Hyperparathyroidism: Enhanced Conversion Into 1,25‐Hydroxyvitamin D May Not Be “True” Deficiency

**DOI:** 10.1002/jbm4.10415

**Published:** 2020-10-18

**Authors:** Udaya M Kabadi

**Affiliations:** ^1^ Division of Endocrinology, Department of Medicine, Broadlawns Medical Center, Des Moines, IA University of Iowa Iowa City IA USA

**Keywords:** 1,25 OH VITAMIN D, VITAMIN D, VITAMIN D DEFICIENCY, PRIMARY HYPERPARATHYROIDISM, PARATHYROID HORMONE

## Abstract

Vitamin D deficiency is reported in individuals with primary hyperparathyroidism (PHP). However, decreased 25OHD may be attributed to enhanced conversion into 1,25‐hydroxyvitamin D [1,25(OH)D]. To examine vitamin D metabolism in individuals with PHP, serum calcium, PTH, 25OHD, and 1,25(OH)D levels were determined in 210 adults: 102 with PHP, 40 with normal 25OHD, and 68 with vitamin D deficiency. Concentrations were redetermined in 37 individuals with PHP following vitamin D supplementation and 43 patients postsurgery. Comparisons were conducted by Student's *t* test and ANOVA. Correlations were assessed between PTH and 25OHD, 1,25(OH)D, and 1,25(OH)D/25OHD in individuals with PHP. Calcium, PTH, and 1,25(OH)D were higher (*p* < 0.001) in individuals with PHP (11.4 ± 0.4, 116 ± 21, 79 ± 6) than in individuals with normal 25OHD (9.6 ± 0.2, 49 ± 5, 57 ± 6) and vitamin D deficiency (9.3 ± 0.2, 62 ± 6, 32 ± 4). Compared with individuals with normal 25OHD (47 ± 5), 25OHD was lower (18 ± 3), but not different from subjects with vitamin D deficiency (15 ± 2). In individuals with PHP, vitamin D2 supplementation induced rises in 1,25(OH)D and calcium without lowering PTH, whereas postsurgery, calcium, PTH, 25OHD, and 1,25(OH)D normalized. Finally, in individuals with PHP, significant correlations (*p* < 0.01) were documented between PTH and calcium (*r* = 0.74), 25OHD (*r* = −0.43), 1,25(OH)D (*r* = 0.52), and 1,25(OH)D/25OHD (*r* = 0.46); and between 1,25(OH)D/25OHD and calcium (*r* = 0.47). Subnormal 25OHD in most individuals with PHP may be attributed to enhanced conversion to 1,25(OH)D—not “true” vitamin D deficiency—although in some patients, both PHP and vitamin D deficiency coexisted. Moreover, vitamin D supplementation exaggerated hypercalcemia in individuals with PHP. © 2020 The Author. *JBMR Plus* published by Wiley Periodicals LLC on behalf of American Society for Bone and Mineral Research.

## Introduction

Subnormal serum 25OHD concentrations are reported to occur frequently in individuals with primary hyperparathyroidism (PHP) and are attributed to concurrent vitamin D deficiency.^(^ ^1^, ^2^, ^3^, ^4^, ^5^, ^6^, ^7^, ^8^, ^9^, ^10^, ^11^, ^12^ ^)^ It is plausible that the prevalence of vitamin D deficiency is overestimated and exaggerated because low serum 25OHD levels may be explained by its enhanced conversion into 1,25‐hydroxyvitamin D [1,25(OH)D],

induced by high levels of circulating PTH, a well‐established physiologic mechanism.^(^ ^13^, ^14^ ^)^ Moreover, 1,25(OH)D concentrations in individuals with PHP have been rarely reported in the past 30 years.^(^ ^15^, ^16^, ^17^ ^)^ Therefore, serum concentrations of PTH, calcium, 25OHD, and 1,25(OH)D were determined in individuals with a confirmed diagnosis of PHP. Relationships between these various parameters were examined as well. The study was approved by local institutional review board (Broadlawns Medical Center) and subjects provided informed consent prior to enrollment.

## Subjects and Methods

Serum concentrations of calcium, PTH, 25OHD, and 1,25(OH)D were determined after an overnight fast in 102 consecutive adult subjects: 45 men and 57 women aged 39 to 78 years (49 ± 6) with an established diagnosis of PHP from January 1, 2011 through December 31, 2018. The diagnosis of PHP was documented by persistent elevated serum calcium >10.2 mg/dL (normal range, 8.5 to 10.2) and elevated or inappropriately normal PTH of 55 to 210 U/mL (normal range, 15 to 65) on more than one occasion, as well as 24‐hour urinary calcium excretion of >100 mg. Subjects with a syndrome of familial hypocalciuric hypercalcemia were excluded based on 24‐hour urinary calcium excretion <100 mg. Bone densities were determined in all subjects with PHP to establish eligibility for surgical intervention. There were 67 subjects deemed eligible for surgery based on the presence of clinical manifestations and the presence of two out of three laboratory determinations: (i) serum calcium >11.1 mg/dL, (ii) elevated 24‐hour urinary calcium excretion >200 mg, or (iii) osteoporosis or osteopenia documented by BMD. For comparison, 40 age‐matched (49 ± 4 years) healthy volunteers, 18 men and 22 women, with normal serum concentrations of calcium (8.5 to 10.2 mg/dL), PTH, and 25OHD concentrations (30 to 80 ng/dL) were recruited. Comparisons were also conducted between subjects with PHP and 68 age‐matched subjects, 29 men and 39 women (50 ± 4 years), with vitamin D deficiency. The diagnosis of vitamin D deficiency or insufficiency was established by serum vitamin D concentration <30 ng/dL and low or normal serum calcium as per the guidelines of the Endocrine Society.^(^ ^18^ ^)^ In addition, serum concentrations were reassessed in 80 subjects with PHP: (i) 43 subjects, 19 men and 24 women, following surgery at 3 to 6 months—although 67 subjects were deemed eligible for surgery, 10 opted frequent monitoring instead of surgery and the remaining 14 were treated with the cacimimetic drug, cinacalcet because of age >75 years or <75 years with multiple comorbidities; and (ii) 37 subjects, 18 men and 19 women, who, after 3 months of biweekly supplementation with 50,000 units of vitamin D2 (ergocalciferol; as reported previously,^(^ ^19^ ^)^ had persistent elevated levels of PTH and subnormal levels of 25OHD, despite normalization of serum calcium levels after surgery. Thus, in this group, serum calcium, PTH, 25OHD, and 1,25(OH)D concentrations were determined three times: prior to surgery, postsurgery, and again following vitamin D2 supplementation. Comparisons between groups for all determinations were conducted by Student's *t* test and ANOVA. Multiple correlations were also assessed by linear regression analyses in subjects with PHP first between serum PTH levels and then between concentrations of calcium, 25OHD, and 1,25(OH)D, as well as 1,25(OH)D/25OHD ratios. Correlations were also determined between serum levels of 1,25(OH)D and 1,25(OH)D/25OHD ratios and serum calcium concentrations. The 1,25(OH)D/25OHD ratio is an accepted index of expression of 1,25(OH)D generation from 25OHD.^(^ ^3^, ^9^, ^12^, ^14^ ^)^ PTH and both vitamin D levels were determined by a commercial laboratory (Mayo Clinic, Rochester, MN, USA) using well‐established immunoassays with interassay and intra‐assay coefficients of variation ranging from 12% to 15%. The usual blood tests for calcium, phosphorus, liver enzymes, albumin, serum urea nitrogen, creatinine, electrolytes, glucose, etc., were assessed by the local hospital laboratory using a chemical analyzer.

## Results

Serum calcium, PTH, and 1,25(OH)D concentrations were significantly higher in subjects with PHP when compared with values in heathy volunteers and subjects with vitamin D deficiency (Table 1). Alternatively, serum 25OHD concentration in subjects with PHP was significantly lower than normal, but not when compared with subjects with vitamin D deficiency (Table 1). In subjects with PHP, at 3 to 6 months following surgery, serum calcium and 1,25(OH)D levels declined to the normal range in all subjects (Table 2). However, two distinct groups evolved on analysis of the results in relation to 25OHD and PTH concentrations. In 25 subjects (group1), both 25OHD and PTH concentrations normalized (Table 3). In the remaining 18 subjects (group 2), 25OHD rose significantly, but remained subnormal (<30 ng/dL), whereas PTH declined though stayed supernormal at >65 U/mL (Table 3). Further assessment of the data prior to surgery revealed significantly greater serum calcium and PTH concentrations in group 2 in comparison with group 1(Table 3). Alternatively, both serum 25OHD and 1,25(OH)D levels were significantly lower in group 2 in comparison with group 1 (Table 3). Furthermore, vitamin D supplementation following surgery in this group normalized both serum 25OHD and PTH levels, while maintaining serum calcium and 1,25(OH)D within the normal range (Table 3).

**Table 1:** Serum PTH, 25OHD, 1,25(OH)D, and 1,25(OH)D/25OHD Ratio in 102 Subjects With PHP, 40 Age‐Matched Normal Subjects, and 68 Subjects With Vitamin D Deficiency

Subjects	PTH Pg/mL	Calcium mg/dL	25OHD ng/mL	1,25(OH)D pg/mL	1,25/25
PHP	128 ± 12*	11.2 ± 0.4*	18 ± 2*	79 ± 5*	4.4 ± 0.3*
Vitamin D deficiency	62 ± 6±	9.1 ± 0.2	15 ± 2**	28 ± 4**	1.9 ± 0.1
Normal	41 ± 5	9.4 ± 0.3	47 ± 5	52 ± 7	1.1 ± 0.1
Normal range	15–65	8.5–10.5	20–80	18–72	

1,25(OH)D = 1,25‐hydroxyvitamin D; 1,25/25 = 1,25(OH)D/25OHD; PHP = primary hyperparathyroidism.

* *p* < 0.01 vs normal subject and subjects with low vitamin D.

** *p* < 0.01 vs normal subjects.

**Table 2:** Serum PTH, 25OHD, 1,25(OH)D, and 1,25(OH)D/25OHD Ratio in 43 Subjects With PHP Presurgery and 3‐ to 6‐Months Postsurgery

Subjects	PTH pg/mL	Calcium mg/dL	25OHD ng/mL	1,25(OH)D pg/mL	1,25/25
Presurgery	117 ± 8	11.3 ± 0.6	17 ± 2	82 ± 6	4.5 ± 0.4
Postsurgery	56 ± 5*	9.3 ± 0.3*	42 ± 5*	54 ± 8*	1.2 ± 0.2*

1,25(OH)D = 1,25‐hydroxyvitamin D; 1,25/25 = 1,25(OH)D/25OHD; PHP = primary hyperparathyroidism.

* Postsurgery in comparison to Presurgery; *p* < 0.01.

**Table 3:** Serum Calcium, PTH, 25OHD, and 1,25(OH)D in Group 1 (PHP Alone) and Group 2 (PHP + Vitamin D Deficiency) Presurgery and Postsurgery, and in Group 2 After Vitamin D2 (Ergocalciferol) Supplementation Postsurgery

Group1	Calcium	PTH	25OHD	1,25(OH)D
Presurgery	11.4 ± 0.7	135 ± 9	18 ± 4	95 ± 9
Postsurgery	9.5 ± 0.4**	54 ± 7**	52 ± 7**	65 ± 6**
Group 2				
Presurgery	10.8 ± 0.6*	155 ± 10*	14 ± 3	78 ± 8*
Postsurgery	9.3 ± 0.4**	77 ± 8*,**	23 ± 4 *,**	62 ± 6 ^*,^**
Post D	9.4 ± 0.3	55 ± 6***	48 ± 5***	68 ± 7***

1,25(OH)D = 1,25‐hydroxyvitamin D; PHP = primary hyperparathyroidism; Post D = vitamin D2 (ergocalciferol) supplementation postsurgery.

* Group 2 vs group 1; *p* < 0.05.

** Postsurgery vs presurgery in same group; *p* < 0.0.

*** Post D vs postsurgery in group 2; *p* < 0.05.

In 37 subjects with PHP and subnormal 25OHD levels prior to surgery, weekly administration of 50,000 units of vitamin D2 normalized serum 25OHD levels. However, both calcium and 1,25(OH)D levels rose further, with no significant lowering of serum PTH concentrations (Table 4).

**Table 4:** Serum PTH, 25OHD, 1,25(OH)D, and 1,25(OH)D/25OHD Ratio in 37 Subjects With Primary Hyperparathyroidism Prior to Supplementation and 3 to 6 Months After Weekly Supplementation With 50,000 Units of Vitamin D (Ergocalciferol)

	PTH pg/mL	Calcium mg/dL	25OHDng/mL	1,25(OH)D pg/mL	1,25/25
Prior to supplementation	109 ± 12	10.9 ± 0.3	16 ± 3	70 ± 6	4.4 ± 0.6
After supplementation	102 ± 11	11.4+ 0.4*	45 ± 7*	83 ± 10*	2.3 ± 0.4*

1,25(OH)D = 1,25‐hydroxyvitamin D; 1,25/25 = 1,25(OH)D/25OHD.

* *p* < 0.05 vs prior to supplementation.

Significant positive correlations were documented in subjects with PHP between serum PTH levels, and serum calcium, 1,25(OH)D, and 1,25(OH)D/25OHD ratios (Table 5). Positive correlations were also noted between serum 1,25(OH)D levels and 1,25(OH)D/25OHD ratios, and serum calcium concentrations (Table 5). Finally, a significant negative correlation was observed between serum PTH levels and 25OHD concentrations (Table 5).

**Table 5:** Coefficients of Variation (*r*) for Linear Regression Analysis Between Various Serum Concentrations in the Whole Cohort of Subjects With Primary Hyperparathyroidism

Correlations	*r* Value	*p* Value
PTH vs calcium	0.74	<0.01
PTH vs 25OHD	−0.43	<0.01
PTH vs 1,25(OH)D	0.52	<0.01
PTH vs 1,25/25	0.46	<0.01
1,25(OH)D vs calcium	0.36	<0.05
1,25/25 vs calcium	0.47	<0.01

1,25(OH)D = 1,25‐hydroxyvitamin D; 1,25/25 = 1,25(OH)D/25OHD.

## Discussion

This study documents low serum 25OHD and elevated 1,25(OH)D concentrations in subjects with PHP and hypercalcemia. The presence of low serum 25OHD in subjects with PHP is well‐established.^(^ ^1^, ^2^, ^3^, ^4^, ^5^, ^6^, ^7^, ^8^, ^9^, ^10^, ^11^, ^12^ ^)^ However, concurrently elevated serum 1,25(OH)D concentrations in subjects with PHP have rarely been reported in the past 30 years.^(^ ^15^, ^16^, ^17^ ^)^ This study also found normalization of mean concentrations of serum calcium, 25OHD, and 1,25(OH)D following a decline in circulating serum PTH levels after surgery.

However, on further examination of postsurgical data, normalization of these levels did not ensue in all individuals with PHP. Two distinct populations evolved: (i) 25 subjects with normalization of serum calcium, PTH, 25OHD, and 1,25(OH)D (group 1); and (ii) 18 subjects (group 2) with normalization of serum calcium and 1,25(OH)D levels, but improvement without normalization of 25OHD and PTH concentrations. Persistent subnormal 25OHD levels and elevated PTH concentrations may indicate a presurgical presence of both PHP and vitamin D deficiency in this group. Moreover, the presence of both disorders in this group is further evident as documented by significantly lower serum calcium, 25OHD, and 1,25(OH)D levels with a significantly higher PTH concentration in this group when compared with group 1 prior to surgery. The significantly higher PTH concentration in this group may also be attributed to the concurrent presence of both secondary hyperparathyroidism induced by vitamin D deficiency as well as PHP. Alternatively, hypercalciuria may have contributed to persistently elevated PTH levels. However, normalization of both 25OHD and PTH levels, following the administration of vitamin D2 over 4 to 6 months after surgery in this group, may indicate a minimal contribution of hypercalciuria in the persistent elevation of PTH levels, and confirms the presence of both disorders prior to surgery.

This study also found a further rise in serum calcium on normalization of serum 25OHD consistent with most previous reports.^(^ ^1^, ^2^, ^6^, ^15^, ^16^, ^17^, ^20^, ^21^ ^)^ In contrast, an isolated study has documented a lack of exaggerated rise in already existent hypercalcemia upon vitamin D supplementation.^(^ ^12^ ^)^ However, in this study, 25OHD remained subnormal probably because of inadequate supplementation. The rise in serum calcium in our study, and in others, may be attributed to a further exaggerated rise in 1,25(OH)D and a lack of suppression or a paradoxical rise of elevated PTH. This finding is consistent with a recent report documenting the onset of hypercalcemia following vitamin D administration in subjects with normocalcemic PHP caused by a lack of suppression of PTH.^(^ ^22^ ^)^ However, this study's finding regarding 1,25(OH)D and PTH following administration of vitamin D2 in subjects with PHP has not been previously reported in the literature.

The significant negative correlation between PTH and 25OHD found in this study is consistent with that reported in the literature.^(^ ^3^, ^5^, ^8^, ^10^ ^)^ However, the data regarding PTH, 1,25 OH Vitamin D and 1,25 /25 OH vitamin D ratio on one aspect and serum calcium on the other is sparse.^(^ ^17^ ^)^ Significant positive correlations between serum PTH concentrations on one aspect and 1,25(OH)D levels and 1,25(OH)D/25OHD ratios may indicate the role of PTH in the increased generation of 1,25(OH)D from 25OHD. However, the greater correlation between PTH and serum calcium compared with 1,25(OH)D and serum calcium confirms PTH as the primary contributor to hypercalcemia. The major role of PTH may be attributed to its well‐established, multiple direct physiologic effects on calcium metabolism, including enhanced gastrointestinal absorption and bone resorption, as well as inhibited renal excretion.^(^ ^12^, ^13^ ^)^ However, PTH induced a rise in 1,25(OH)D via accelerated conversion of 25OHD; this may also be a minor contributor to hypercalcemia because calcium absorption by the gut and the kidneys is facilitated by 1,25(OH)D. The minor contribution of 1,25(OH)D to hypercalcemia was also evident by an initial transient decline in serum calcium upon administration of phosphate, probably via inhibition of renal generation of 1,25(OH)D from 25OHD.^(^ ^23^, ^24^, ^25^ ^)^ However, a consequent exaggerated rise in serum PTH with induction of a recurrence of hypercalcemia may confirm the major role of PTH in hypercalcemia.

Therefore, low serum 25OHD in subjects with PHP alone (group1) was caused by enhanced conversion into 1,25(OH)D induced by elevated circulating PTH, a well‐established physiologic effect of PTH and not a “true” vitamin D deficiency. This pathophysiologic mechanism was further evident by normalization of both vitamin D fractions following surgery. Finally, concurrent vitamin D deficiency did appear to exist in some subjects with PHP (group 2), leading to a lesser degree of hypercalcemia and 1,25(OH)D elevation despite a greater PTH concentration in comparison with group 1. An exacerbation of hypercalcemia in subjects with PHP occurred on normalization of serum 25OHD levels following the administration of vitamin D2, probably caused by a further rise in serum 1,25(OH)D levels via increased generation induced by persistently elevated circulating PTH.

### PEER REVIEW

The peer review history for this article is available at https://publons.com/publon/10.1002/jbm4.10415.
